# Effects of Biochar on Cadmium Availability, Nitrification and Microbial Communities in Soils with Varied pH Levels

**DOI:** 10.3390/microorganisms13040839

**Published:** 2025-04-07

**Authors:** Wei Zhao, Xiaoxu Cao, Hong Pan, Yanhong Lou, Hui Wang, Quangang Yang, Yuping Zhuge

**Affiliations:** National Engineering Research Center for Efficient Utilization of Soil and Fertilizer Resources, College of Resources and Environment, Shandong Agricultural University, Tai’an 271018, China; zhao05618@163.com (W.Z.); cxx15930719505@163.com (X.C.); yanhonglou@sdau.edu.cn (Y.L.); huiwang@sdau.edu.cn (H.W.); sttzzy@sdau.edu.cn (Q.Y.)

**Keywords:** cadmium (Cd) contamination, soil pH, biochar, ammonia-oxidizing microorganisms, nitrification, soil remediation

## Abstract

Cadmium (Cd) contamination poses severe threats to agricultural productivity and ecosystem health. Biochar has shown promise in immobilizing Cd and enhancing microbial functions, yet its pH-dependent mechanisms remain underexplored. This study aimed to elucidate pH-dependent variations in biochar-mediated cadmium (Cd) immobilization efficiency, nitrification activity, and bacterial community diversity across soils of contrasting pH levels, with mechanistic insights into the synergistic interplay between biochar properties and soil pH. Real-time quantitative PCR (qPCR) and high-throughput sequencing were used to investigate the effects of a 1% (*w/w*) biochar amendment on ammonia-oxidizing microorganism abundance and microbial diversity in neutral Shandong soil (SD, pH 7.46) and acidic Yunnan soil (YN, pH 5.88). In neutral SD soil, available Cd decreased from 0.22 mg kg^−1^ (day 0) to 0.1 mg kg^−1^ (day 56) and stabilized, accompanied by insignificant changes in ammonia-oxidizing bacteria (AOB) abundance. However, nitrification activity was enhanced through the enrichment of *Nitrospira* (nitrite-oxidizing bacteria within Nitrospirales and Nitrospiraceae). In acidic YN soil, biochar reduced available Cd by 53.37% over 56 days, concurrent with a 34.28% increase in AOB *amoA* gene abundance (predominantly *Nitrosomonadales*), driving pH-dependent nitrification enhancement. These findings demonstrated that biochar efficacy was critically modulated by soil pH; the acidic soils require higher biochar dosages (>1% *w/w*, adjusted to local soil properties and agronomic conditions) for optimal Cd immobilization. Meanwhile, pH-specific nitrifier taxa (*Nitrosomonadales* in acidic vs. *Nitrospira* in neutral soils) underpinned biochar-induced nitrification dynamics. The study provided a mechanistic framework for tailoring biochar remediation strategies to soil pH gradients, emphasizing the synergistic regulation of Cd immobilization and microbial nitrogen cycling.

## 1. Introduction

Soil heavy metal residue has remained a focal research topic in environmental conservation. According to the National Soil Pollution Status Bulletin, cadmium (Cd) contamination was particularly high in industrial and mining soils. The high toxicity, high mobility, and difficult degradation of Cd lead to persistent environmental risks [1]. Acting as an environmental sink, Cd enters soils through industrial emissions and mining activities, migrates via the soil-plant system, and creates a biomagnification effect. Ahmed et al. [2] (pp. 836–851) highlighted that Cd contamination from wastewater irrigation poses significant risks, with crops such as cereals in affected regions demonstrating substantially higher heavy metal content compared to freshwater-irrigated areas. In grains cultivated near mining areas in China, 14.4–27.8% of the sampled crops exceeded the maximum permissible limit (MPL) of 0.2 mg kg^−1^ for Cd [3], thus directly threatening food security and human health.

The heterogeneity of soil physicochemical properties (e.g., pH) significantly influenced Cd dynamics in soils, leading to substantial variations in Cd solubility and toxic potential. Alkaline soils exhibited lower Cd mobility and bioavailability compared to acidic soils, primarily due to pH-dependent immobilization mechanisms. During soil oxidation, exchangeable Cd is transformed into stable precipitates (e.g., iron–manganese oxides, carbonates) under alkaline conditions, which could effectively reduce the environmental availability of Cd [4]. Relative to neutral soils in northern China, southern soils with lower pH favor metal dissolution and exhibit higher redox activity [5]. Notably, the geochemical mobilization of Cd exhibited significant enhancement, a phenomenon consistently associated with profound perturbation of edaphic biogeochemical processes, particularly nitrification metabolism. As an essential biogeochemical node in the global nitrogen cycle, nitrification dynamics are intrinsically regulated by microbial nitrogen-transforming consortia through enzymatic catalysis [6]. Cd exposure imposes suppressive effects on ammonia monooxygenase (AMO) catalytic activity via competitive inhibition of metalloenzyme active sites, thereby disrupting nitrifier-mediated processes involving both ammonia-oxidizing archaea (AOA) and bacteria (AOB) [7]. Quantitative molecular analyses revealed that 0.4 mmol kg^−1^ Cd loading significantly depressed *amoA* gene abundance in AOA and AOB populations within luvisols [8]. Complementary fluvisol microcosm experiments demonstrated dose-responsive suppression of potential nitrification rates (PNR), confirming the pan-edaphic nature of Cd inhibition.

This pH-dependent speciation dichotomy necessitates differential remediation approaches [9]. Acidic soils require geochemical stabilization through pH elevation via proton consumption through alkaline mineral dissolution, while neutral soils demand enhanced chemisorption through surface ligand optimization. X-Ray absorption fine structure (XAFS) analyses confirmed that carboxyl (-COOH) and hydroxyl (-OH) groups dominate Cd complexation in neutral matrices. However, the remediation paradigm extends beyond abiotic mechanisms. Biotic mediation through phyto-rhizosphere interactions, detritivore bioturbation, and microbial redox transformations collectively contributes to ecological stabilization [10]. Notably, biochar amendment stimulates the proliferation of metal-resistant taxa, including Rhodocyclaceae, Geobacter, and Burkholderia spp., as revealed by 16S rRNA sequencing [11,12]. In particular, *Burkholderia* spp. (Proteobacteria) mediates sulfate-activated biomineralization, precipitating crystalline Ca_10−x_Cd_x_(PO_4_)_6_(OH)_2_ phases through enzymatically controlled nucleation processes [12].

Biochar, an organic carbon-rich amendment characterized by high specific surface area and porous structure, demonstrates promising potential in soil remediation [13]. Biochar can neutralize soil acidity, improve physicochemical properties, and modulate microbial community structure, thereby fostering a habitat conducive to functional microorganisms [14]. Simultaneously, biochar contains abundant oxygen-containing functional groups, such as carboxyl and hydroxyl groups, which immobilize Cd through complexation and ion exchange reactions, effectively fixing Cd onto the biochar surface [15]. Furthermore, biochar facilitated Cd fixation by regulating iron and sulfur redox cycles, which synergistically reduced Cd mobility [16], further diminishing Cd bioavailability. Beyond metal immobilization, biochar mitigated nitrogen loss by adsorbing ammonium and nitrate within its porous matrix, thereby stabilizing nitrification-denitrification dynamics and enhancing overall soil ecosystem resilience. These findings collectively underscore the imperative for pH-stratified biochar engineering, where optimal performance requires precise coordination of surface chemistry, microbial ecology, and chemodynamic coordination effects across soil matrices.

The porous structure of biochar facilitates the “capture” of ammonium and nitrate via adsorption, minimizing nitrogen loss and enhancing nitrogen retention, thereby regulating soil nitrogen transformation processes [17]. In soil nitrification, ammonia-oxidizing archaea (AOA) and bacteria (AOB) function synergistically. However, studies have shown inconsistent effects of biochar on AOA and AOB abundances. For instance, in coastal saline-alkali soils, exogenous biochar addition reduced the relative abundance of AOA but increased that of AOB [18], whereas in acidic soils, both AOA and AOB abundances increased following biochar amendment, highlighting the critical role of soil pH in determining ammonia oxidation processes. Previous research primarily focused on the effects of exogenous amendments on nitrification and bacterial abundance in contaminated soils, with limited understanding of how nitrification processes and ammonia-oxidizing microorganisms respond to varying pollution stresses. To address this, two Cd-contaminated soils with distinct pH levels were collected for controlled incubation experiments with a 1% (*w/w*) biochar amendment. Real-time qPCR and high-throughput sequencing were employed to investigate soil nitrification dynamics and microbial responses. The objectives of this study were (1) to investigate the remediation efficacy of biochar in the two contaminated soils, (2) to elucidate functional microbial differences between neutral Shandong soil (SD) and acidic Yunnan soil (YN), and (3) to explore partial interactions between biochar-mediated nitrification processes and Cd passivation mechanisms in both soils.

## 2. Materials and Methods

### 2.1. Sampling Site

Soil samples were collected from agricultural soils in Shandong and Yunnan Province, China, in November 2019. The sampling sites showed a well-developed vegetation cover, with both trees and herbaceous species. The Shandong soil sample was collected adjacent to an abandoned chemical factory site located in a warm-temperate semi-arid climate zone (mean annual temperature: 13.3 °C; mean annual precipitation: 610 mm), classified as brown soil with a DTPA-extractable cadmium (available Cd) content of 0.37 mg kg^−1^. The Yunnan soil was collected from historical Pb-Zn mining areas in a subtropical monsoon climate zone (mean annual temperature: 18 °C; annual precipitation: 800–2000 mm), categorized as red soil with an available Cd content of 1.57 mg kg^−1^. Five surface soil samples (0–20 cm depth) were randomly collected and homogenized into one composite sample per location, denoted as SD (Shandong) and YN (Yunnan). Soil samples were transported on ice to the laboratory immediately after sampling and were then homogenized through a 2-mm sieve. A portion of the soils was used to analyze the physicochemical properties (Table 1), and the remainder of the soil was stored at 4 °C for the incubation experiment.

### 2.2. Construction and Sampling of Soil Microcosms

The cottonseed hull biochar (BC) was selected as the soil amendment for its superior performance in heavy metal immobilization due to oxygen-functional group enrichment [19]. Cottonseed husks were produced in Linyi, Shandong Province. The cottonseed hulls were prepared by air-drying the raw material at ambient temperature, followed by pyrolysis in a nitrogen-flushed muffle furnace (Lindberg, Type 51662-HR, Watertown, WI, USA) at 350 °C (1600 mL min^−1^ N_2_ flow) for 4 h. The resultant BC was sieved through a 100-mesh sieve prior to use. Key physicochemical properties are presented in Table 2.

The soil samples (equivalent to 50 g dry weight) were pre-incubated at 40% field moisture capacity in a 250 mL serum bottle for five days at 25 °C in the dark before the incubation experiment. After pre-incubation, BC at 1% (*w/w*, 0.5 g) was thoroughly mixed with soil from Shandong (SD) and Yunnan (YN), respectively. Approximately 50 μg urea-N g^−1^ dry weight soil (d.w.s) was added through dropwise addition of a freshly made urea solution to achieve 60% of the maximum water-holding capacity. Microcosms were established using four treatments (each in triplicate), including (I) SD-CK (Shandong control soil), (II) YN-CK (Yunnan control soil), (III) SD-BC (Shandong soil + BC), and (IV) YN-BC (Yunnan soil + BC).

Homogenized soil samples were incubated in darkness at 25 °C for 8 weeks. Bottles were sealed with Parafilm-M to maintain humidity while punctured with uniform micropores to ensure aerobic conditions. Soil moisture was monitored gravimetrically. Destructive sampling occurred at 0, 7, 28, and 56 days. About 2 g of soil from each triplicate microcosm was transferred immediately to a −80 °C freezer for molecular analysis. The remaining soil was used to analyze soil inorganic nitrogen and available Cd content.

### 2.3. Soil and Biochar Sample Analysis

Soil pH and electrical conductivity (EC) were determined in a solution suspension of soil and distilled water at ratios of 1:2.5 and 1:5 using a Sartorius basic pH meter (Sartorius Scientific Instruments Co. Ltd., Beijing, China) and a digital conductivity meter (Crison 522, Barcelona, Spain), respectively [20]. The soil organic matter (SOM) content was measured by wet digestion with H_2_SO_4_-K_2_Cr_2_O_7_ [20]. Available P (AP) was extracted using sodium bicarbonate and determined using the molybdenum blue method [20]. Available K (AK) was extracted using ammonium acetate and determined by flame photometry [20]. Soil was homogenized with 1 M KCl (soil/KCl, 1:10) by shaking at 250 rpm for 30 min and then passed through filter paper for the determination of NH_4_^+^-N and NO_3_^−^-N using a Skalar SAN Plus (Skalar Inc., Breda, The Netherlands) segmented flow analyzer [21]. Soil particle size distribution was determined using the rapid sieving method described by Kettler et al. [22] (pp. 849–852). Available Cd was extracted with diethylenetriaminepentaacetic acid (DTPA) following the atomic absorption spectroscopy protocol GB/T 23739-2009 [23]. Filtered extracts were analyzed using inductively coupled plasma optical emission spectrometry (ICP-OES, Thermo Fisher, Altrincham, UK). Soil samples were digested with aqua regia to determine the concentrations of total Cd using inductively coupled plasma mass spectrometry (ICP-OES; Agilent, Santa Clara, CA, USA) using the environmental standard (HJ 803-2016) [24]. The contents of C, H, N, and S in biochar were measured using an elemental analyzer (vario macro cube, Elementar, Langenselbold, Germany), and the O content was determined by means of difference calculation [19]. The ash and volatile matter content of biochar were determined by heating in a muffle furnace to a constant weight, followed by cooling in a desiccator [25].

### 2.4. DNA Extraction and Illumina MiSeq Sequencing

DNA extractions were carried out in triplicate for all samples using the FastDNA SPIN Kit for Soil (MP Biomedicals, LLC, Solon, OH, USA). The quantity and purity of the extracted DNA were estimated and verified by NanoDrop^®^ ND-2000 spectrophotometer (NanoDrop Technologies, Wilmington, DE, USA).

AOA and AOB amoA gene abundance were quantitatively analyzed using real-time fluorescence quantitative PCR. The measurement instrument was a fluorescence quantitative instrument (ABI 7500 Real-Time PCR System; Thermo Fisher Scientific, Waltham, MA, USA), and the primer sequences are presented in Table 3.

The V4 region of the bacterial 16S rRNA gene was amplified using the primers 338F (5′ACTCCTACGGGAGGCAGCAG3′) and 806R (5′GGACTACHVGGGTWTCTAAT3′) on an ABI GeneAmp^®^ 9700. Three negative controls (extraction blanks with no soil added) were processed in parallel to detect potential contamination during DNA extraction. To validate PCR specificity and efficiency, a positive control (ZymoBIOMICS Microbial Community Standard, Zymo Research, Irvine, CA, USA) and a no-template negative control (NTC) were included in each amplification batch [26]. All amplifications were conducted in a 20 μL mixture including 4 μL 5 × FastPfu Buffer, 2 μL 2.5 mM dNTPs, 0.8 μL forward primer (5 μM), 0.8 μL reverse primer (5 μM), 0.4 μL FastPfu Polymerase, 0.2 μL BSA, and 10 ng template DNA. Amplicons were extracted from 2% agarose gel and purified using an AxyPrep DNA Gel Extraction Kit (Axygen Biosciences, Union City, CA, USA) according to the manufacturer’s instructions. Triplicate DNA extracts were prepared for each soil sample (*n* = 3 per treatment). For technical reproducibility, each DNA extract was subjected to three independent PCR amplifications (technical triplicates), followed by sequencing library preparation. The coefficient of variation (CV) of microbial abundances across technical triplicates was calculated, with CV values < 5% confirming high intra-sample consistency. Purified products were pooled in equimolar amounts and paired-end sequenced (2 × 300) on an Illumina MiSeq platform (Majorbio Bioinformatics Technology Co., Ltd., Shanghai, China) according to the standard protocols. Raw FASTQ files were demultiplexed and quality-filtered using QIIME (version 1.17) using the criteria described in Pan et al. [27] (p. 74). Negative control samples yielded < 100 reads and were excluded from downstream analyses. High-quality sequences were clustered into operational taxonomic units (OTUs) at ≥97% similarity using the UPARSE pipeline (v7.0.1090, http://drive5.com/uparse/ (accessed on 30 March 2022)) [28].

**Table 3:** Quantitative PCR and primers.

Target Genes	Primer Name	Primer Sequence (5′–3′)	Reaction Condition	Reference
Archaeal *amoA*	Arch-*amoA*F	STAATGGTCTGGCTTAGACG	95 °C, 3 min; 38 × (95 °C, 20 s; 55 °C, 30 s; 72 °C, 45 s); Melt curve 60 °C to 95 °C, increment 0.5 °C	Francis et al. [29] (pp. 14683–14688)
	Arch-*amoA*R	GCGGCCATCCATCTGTATGT		
Bacterial *amoA*	*amoA*-1F	GGGGTTTCTACTGGTGGT	95 °C, 3 min; 38 × (95 °C, 20 s; 56 °C, 30 s; 72 °C, 30 s); Melt curve 60 °C to 95 °C, increment 0.5 °C	Rotthauwe et al. [30] (pp. 4704–4712)
	*amoA*-2R	CCCCTCKGSAAAGCCTTCTTC		

### 2.5. Statistical Analysis

One-way analysis of variance followed by Duncan’s multiple range test was used to check for significant differences between treatments for soil mineral nitrogen contents, DTPA-extractable Cd, and α-diversity indices using SPSS software (version 24). *p* < 0.05 was used to denote statistical significance. Alpha diversity indices (Shannon, Simpson, PD, Chao1, and Ace) were calculated using Mothur (v1.30.2, https://mothur.org/wiki/calculators/ (accessed on 30 May 2024)). Beta diversity analysis, based on UniFrac distances, and principal component analysis (PCA) were performed using the “vegan” package in R (v3.5.1). Linear discriminant analysis effect size (LEfSe) (http://huttenhower.sph.harvard.edu/LEfSe/ (accessed on 2 June 2024)) was performed to identify taxa differentially responding to biochar addition. The LDA threshold was set to 3 (*p* < 0.05). Functional prediction of 16S rRNA gene sequences was conducted via PICRUSt1, annotated against the COG database to infer metabolic pathway abundances and functional profiles across samples.

## 3. Results

### 3.1. Effects of Biochar Addition on Soil Available Cd in Two Soils with Varied pH

**Figure 1:**
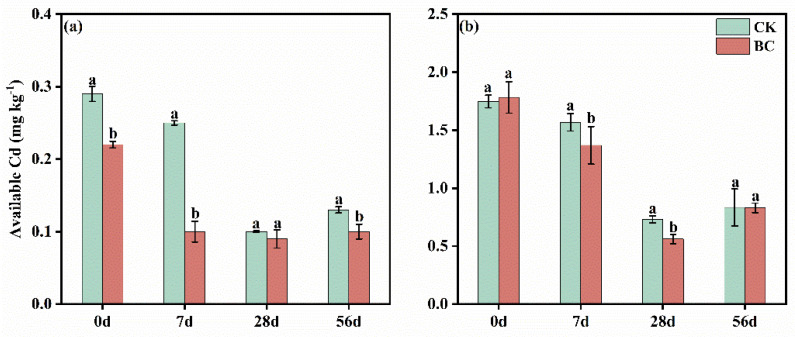
Changes in the content of available Cd in SD (**a**) and YN (**b**) soils with and without biochar addition. Note: Different letters indicated significant differences among treatments (*p* < 0.05), the same below.

In SD-CK soils, available Cd decreased from 0.29 mg kg^−1^ on day 0 to 0.25 mg kg^−1^ on day 7 and further to 0.10 mg kg^−1^ on day 28 and finally increased to 0.13 mg kg^−1^ on day 56 (Figure 1a). In contrast, the available Cd content in SD-BC soils sharply decreased to 0.10 mg kg^−1^ on day 7 and remained relatively stable (0.09–0.10 mg kg^−1^) throughout the subsequent incubation period (days 7–56). As for the YN-CK soils, available Cd content declined from 1.75 mg kg^−1^ on day 0 to 1.57 mg kg^−1^ on day 7 and 0.73 mg kg^−1^ on day 28, then increased to 0.83 mg kg^−1^ by day 56 (Figure 1b). Biochar amendment significantly decreased available Cd to 0.56 mg kg^−1^ on day 28 and increased to 0.83 mg kg^−1^ on day 56.

### 3.2. Effects of Biochar Addition on Nitrification Activity in Soils with Varied pH

**Figure 2:**
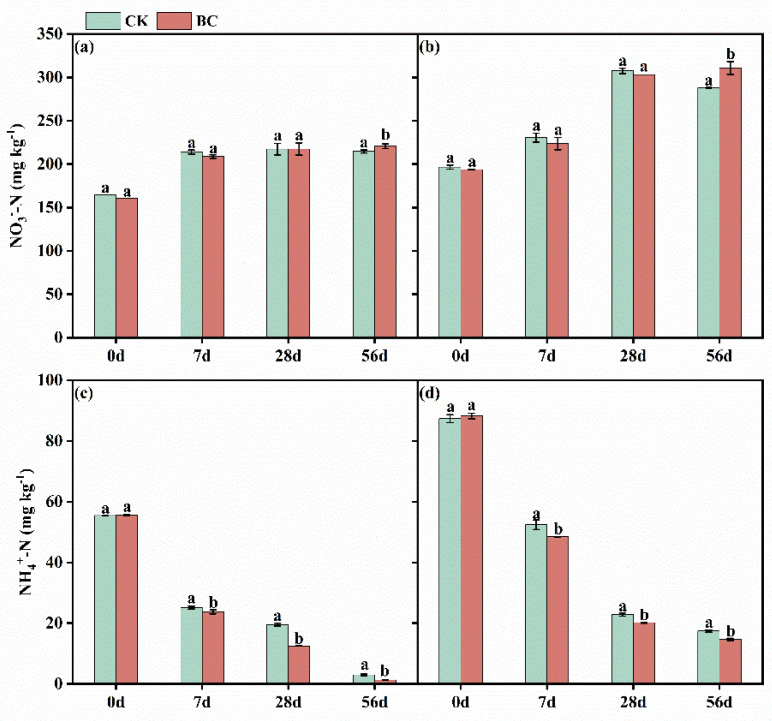
Dynamics of soil inorganic nitrogen content in SD (**a**,**c**) and YN (**b**,**d**) soils with and without biochar addition.

During the incubation, the NO_3_^−^-N concentrations in SD-CK and YN-CK treatment soils reached peak levels of 217.35 and 307.54 mg kg^−1^, respectively, on the 28th day (Figure 2a,b). Conversely, the NO_3_^−^-N concentrations in the SD-BC and YN-BC soils were significantly elevated compared to the control treatments, reaching 220.87 and 310.89 mg kg^−1^, respectively, on day 56. For NH_4_^+^-N, concentrations in SD-CK and YN-CK soils decreased markedly by day 56 compared to day 0, with reductions of 18.66-fold (from 55.47 to 2.97 mg kg^−1^) and 5.01-fold (from 87.38 to 17.45 mg kg^−1^), respectively (Figure 2c,d). Conversely, NH_4_^+^-N concentrations in SD-BC and YN-BC treated soils on day 56 were lower than those in the control treatments, with values of 1.29 and 14.66 mg kg^−1^, respectively.

**Figure 3:**
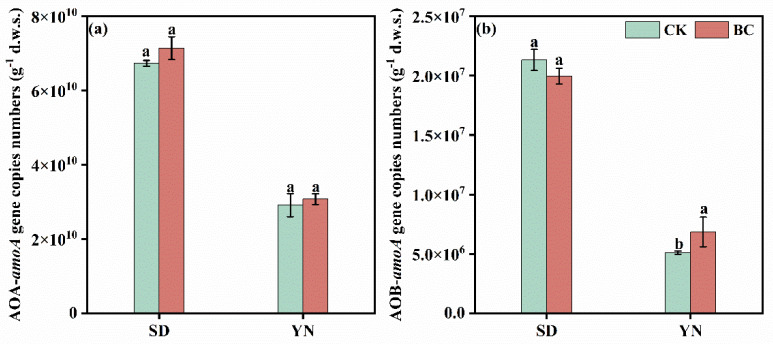
Abundance of soil AOA *amoA* (**a**) and AOB *amoA* (**b**) genes in SD and YN soils with and without biochar addition.

In SD soils, biochar amendment increased the copy numbers of the AOA *amoA* gene, rising from 6.74 × 10^10^ copies g^−1^ d.w.s in the SD-CK to 7.14 × 10^10^ copies g^−1^ d.w.s in the SD-BC treated soil. Conversely, the abundance of the AOB *amoA* gene in SD soil was reduced by biochar addition, declining from 2.13 × 10^7^ copies g^−1^ d.w.s (SD-CK) to 1.99 × 10^7^ copies g^−1^ d.w.s (SD-BC) (Figure 3a,b). In contrast, YN red soil exhibited a divergent response. Biochar addition elevated both AOA and AOB *amoA* gene abundances. The AOA *amoA* gene increased from 2.91 × 10^10^ copies g^−1^ d.w.s (YN-CK) to 3.07 × 10^10^ copies g^−1^ d.w.s (YN-BC), while the AOB *amoA* gene abundance surged from 5.10 × 10^6^ copies g^−1^ d.w.s (YN-CK) to 6.85 × 10^6^ copies g^−1^ d.w.s (YN-BC).

### 3.3. Effects of Biochar Addition on Soil Bacterial Community Diversity in Two Soils with Varied pH

To investigate the diversity and structure of microbial communities, 16S rRNA sequencing was performed using Illumina high-throughput sequencing technology. A total of 906,659 valid sequences were obtained from 18 soil samples, with an average length of 415 bp. Analysis revealed 3178 and 2771 operational taxonomic units in SD and YN soil samples, respectively.

**Figure 4:**
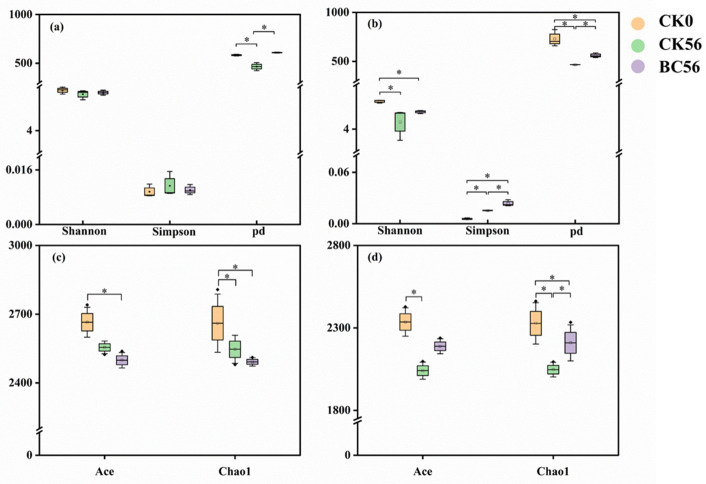
Soil bacterial community α-diversity indices in SD (**a**,**c**) and YN (**b**,**d**) soils with and without biochar addition. The significance levels were as follows: * *p* < 0.05.

In both SD and YN soils, the Shannon and phylogenetic diversity (pd) indices exhibited similar trends in community diversity, with species differences ranked as CK0d > BC56d > CK56d (Figure 4). Nevertheless, in SD-BC56d soil, the richness of bacterial communities experienced a substantial decline after 56 days of incubation and biochar treatment (Figure 4). The Ace and Chao1 indices followed the trend CK0d > CK56d > BC56d (*p* < 0.05), with SD-BC56d exhibiting the lowest richness among all SD samples, yielding Ace and Chao1 indices of 2498.75 and 2491.69, respectively. In the YN soil area, the Simpson index increased significantly (by 1.59%) following biochar amendment in comparison to CK0d, while no significant differences (*p* > 0.05) were observed in SD soil. In contrast, bacterial richness in YN soil samples aligned with the Shannon index trend: CK0d > BC56d > CK56d.

**Figure 5:**
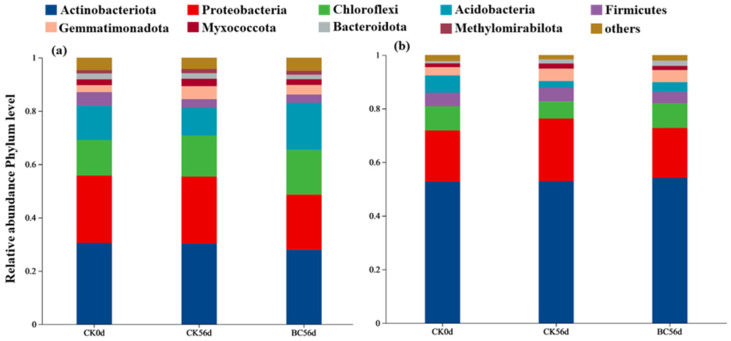
Soil bacterial community composition in SD (**a**) and YN (**b**) soils with and without biochar addition.

**Figure 6:**
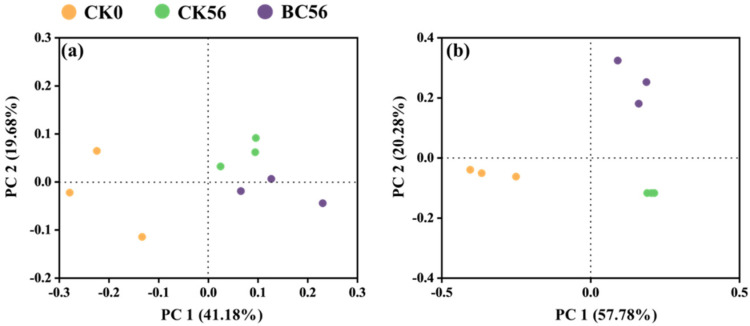
Principal component analysis (PCA) of soil bacterial communities in SD (**a**) and YN (**b**) soils with and without biochar addition.

The bacterial communities in SD and YN soils exhibited marked compositional divergence (Figure 5). In SD soils, the dominant phyla (top five) included Actinobacteria, Proteobacteria, Chloroflexi, Acidobacteria, and Firmicutes, collectively accounting for 84.38–87.12% of total bacterial sequences. In YN soils, these same phyla represented 89.91–92.38% of communities, with Actinobacteria dominating at significantly higher relative abundances (52.79–54.22% across YN-CK0d, YN-CK56d, and YN-BC56d) compared to SD soils (28.00–30.49%). In SD-BC56d treated soils, Proteobacteria abundance decreased by 18.14% (from 25.25% under SD-CK0d treatment to 20.67% under SD-BC56d treatment), Acidobacteria increased by 36.76% (from 12.96% to 17.72%). Conversely, YN-BC56d soils showed a 43.07% reduction in Acidobacteria (from 6.42% in YN-CK0d to 3.66% in YN-BC56d). Notably, Methylomirabilota was uniquely detected in SD soils across all treatments, suggesting region-specific microbial adaptations. PCA based on Euclidean distance confirmed distinct clustering patterns between SD and YN soils (Figure 6). YN soils exhibited greater treatment-induced variability in community structure compared to the more stable SD soil microbiota.

**Figure 7:**
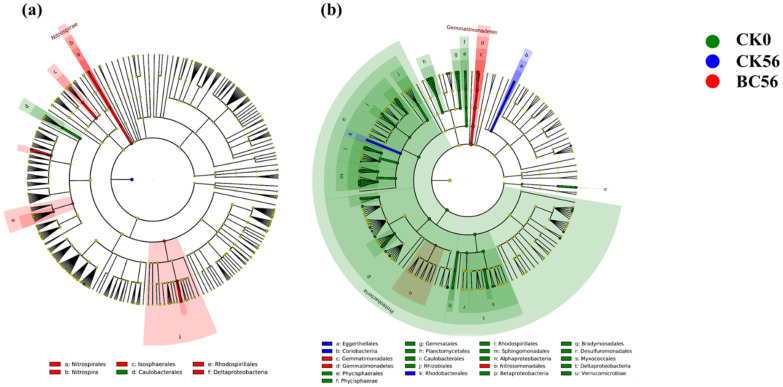
Linear discriminant analysis effect size (LEfSe) spectra of soil bacterial communities in SD (**a**) and YN (**b**) soils with and without biochar addition. Taxa surpassing the LDA threshold (score > 3) were annotated, with cladogram rings denoting taxonomic ranks from phylum (innermost), class, order, family, genus (outermost). Panel labels (a–u) corresponded to bacterial taxa meeting the significance criterion.

LEfSe analysis identified three, one, and fourteen distinct clades or taxa in SD soil under CK0d, CK56d, and BC56d treatments, respectively (Figure 7a). Compared to CK0d and CK56d soils, BC56d treatment significantly increased the relative abundance of *Nitrospira*, Nitrospirales, and Nitrospiraceae (nitrifying spiral bacteria). In YN soil, sixty-two, ten, and seven clades or taxa were identified in CK0d, CK56d, and BC56d treatments, respectively (Figure 7b). Relative to CK56d soil, BC56d treatment reduced the abundance of Actinobacteria and Proteobacteria, while Nitrosomonadales remained relatively high. Additionally, Gemmatimonadetes was significantly enriched in YN-BC56d soil.

### 3.4. Effects of Biochar Addition on Soil Bacterial Community Function in Two Soils with Varied pH

**Figure 8:**
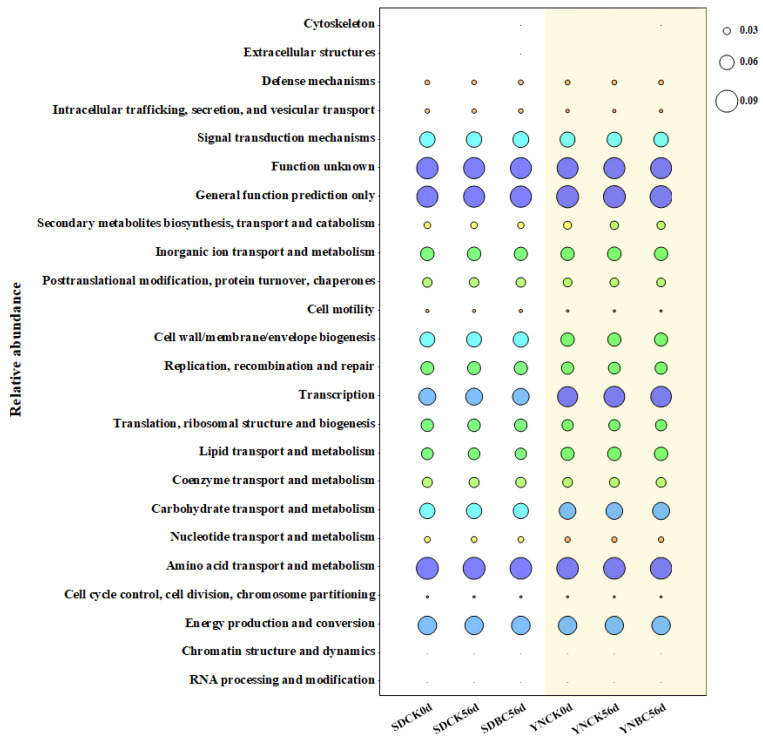
Integration map of the top 20 enriched COG pathways in SD and YN soils with and without biochar addition. The white backgrounds corresponded to SD soils, while light-yellow backgrounds denoted YN soils. The circle corlor was used to distinguish the relative abundance of bacterial functional categories.

Functional predictions based on the COG database revealed distinct patterns in SD and YN soils (Figure 8). In both soil types, CK56d and BC56d treatments exhibited similar functional profiles but differed in the abundance of specific functional categories. The predominant functional categories across all soil samples included Amino acid transport and metabolism, General function prediction only, and Function unknown. In contrast, categories such as Cytoskeleton, Extracellular structures, RNA processing and modification, and Chromatin structure and dynamics were less abundant.

In SD soils, the functional abundance of Coenzyme transport and metabolism was notably enriched following biochar amendment, alongside increased activity in Replication, recombination and repair processes, and Defense mechanisms. Compared to CK56d, SD-BC56d increased these functions by 1.23%, 1.16%, and 3.45%, respectively, while YN-BC56d elevated them by 1.09%, 2.46%, and 1.79%. Notably, the categories Intracellular trafficking, secretion, and vesicular transport and Nucleotide transport and metabolism were more pronounced in SD soils, accounting for 1.81–1.59% and 2.38–2.39%, respectively. In YN soils, functional abundance of Carbohydrate transport and metabolism showed higher relative abundances (4.02–4.06%), alongside increased activity in Secondary metabolites biosynthesis, transport and catabolism (3.39–3.45%) and Transcription processes (8.22–8.49%) than SD soils.

## 4. Discussion

### 4.1. Effects of Biochar Amendment on Cd Availability in Soils with Varied pH

Soil physicochemical heterogeneity, including variations in soil texture, pH, and cation exchange capacity (CEC), regulated ion adsorption–desorption, precipitation–dissolution, and related processes in soils [31]. Studies have demonstrated that biochar reduced Cd availability in both soil types, though its mechanisms differed between neutral and acidic conditions [32,33]. In this experiment, biochar amendment reduced available Cd content in SD-BC (neutral soil) and YN-BC (acidic soil) by day 56 compared to day 0 (Figure 1). However, the stabilization efficiency diverged markedly due to pH-driven interactions.

In neutral SD soil, biochar application improved the soil environment by reducing exchangeable hydrogen, maintaining organic carbon content, and increasing soil porosity [34]. Concurrently, the alkaline properties of biochar elevated soil pH through proton exchange reactions, where alkaline cations released from biochar adsorption sites neutralized soil H^+^. These sites were subsequently occupied by heavy metals such as Cd via complexation mechanisms [33], which likely explains the negative correlation between available Cd and pH in neutral soils of northern China.

In contrast, the temporal fluctuations in Cd availability were observed in acidic YN soil (the available Cd content rose 48.21% from day 28 to 56). This aligned with prior observations of compromised long-term remediation performance of biochar in acidic Cd-contaminated soils [35]. This discrepancy was likely closely linked to the unique physicochemical properties of acidic red soil. First, the release of dissolved salts or colloidal intermediates probably intensified the competition for adsorption sites between soluble Al/Fe (III) and Cd in soil solutions, thereby enhancing Cd solubility [36]. Furthermore, persistent proton flux induced time-dependent ligand protonation dynamics in biochar’s surface functional moieties, progressively diminishing their Cd^2+^ complexation stability constants through competitive cation exchange equilibria [37]. This ligand deactivation mechanism synergistically compromised biochar’s Cd sequestration capacity in acidic soils. Concurrently, accelerated nitrification kinetics during the maturation phase (days 28–56) generated substantial H^+^ flux, as evidenced by micro-electrode profiling, driving soil solution pH below critical ligand deprotonation thresholds and triggering Cd desorption hysteresis [38]. Dose-response analyses revealed threshold limitations of conventional biochar applications, where a 1% (*w/w*) amendment failed to maintain buffering capacity against cumulative acid loading [39]. Longitudinal field trials demonstrated superior performance of 2% (*w/w*) annual re-application, achieving effective proton neutralization through enhanced carbonate dissolution [39]. These findings underscored that chronic acidification fundamentally destabilized biochar-Cd coordination architectures, necessitating adaptive remediation protocols. Strategic co-amendment with alkaline mineral amendments demonstrated synergistic stabilization, simultaneously maintaining optimal nitrifier activity and Cd immobilization efficiency through dual pH buffering and ternary Ca-Cd-CO_3_^2−^ surface precipitation [40]. Implementation of such integrated approaches required precise stoichiometric balancing between acid-neutralizing capacity and biochar’s oxidative aging rate to ensure sustained system resilience.

### 4.2. Effects of Biochar Addition on Nitrification in Soils with Varied pH

Heavy metal Cd contamination has been demonstrated to inhibit soil nitrification in both neutral and acidic soils [41]. Analysis of NO_3_^−^-N and NH_4_^+^-N concentrations showed that the addition of biochar significantly increased NO_3_^−^-N levels, regardless of whether it was SD-BC or YN-BC compared to untreated controls (non-biochar) (Figure 2). This result aligned with findings by Zhao et al. [42] (p. 121631), who reported enhanced nitrification rates in Cd-contaminated acidic soils amended with biochar. The improvement was attributed to the porous structure and oxygen-containing functional groups (e.g., carboxyl, hydroxyl, and phenol groups) in biochar, which could adsorb heavy metal ions and promote their fixation through ion exchange. This reduced Cd interactions with key protein functional groups and alleviating Cd inhibition of nitrifying microorganisms [43]. Mechanistically, Cd pollution suppressed the transcription and activity of AMO, while biochar addition effectively mitigated this suppression [7]. Thus, the observed changes in mineral nitrogen dynamics suggested an indirectly stimulatory effect of biochar on nitrification in Cd-contaminated soils.

In terms of soil nitrifying bacteria, biochar application significantly altered the micro-environments of AOA and AOB communities in neutral and acidic (both YN and SD) soils. Specifically, in acidic YN soil, AOB *amoA* gene abundance increased in YN-BC compared to the untreated control (Figure 3). Jiang et al. [44] (pp. 9–16) demonstrated that AOB community structure and nitrification activity in acidic paddy soils were strongly influenced by soil pH. AOB were sensitive to acidic environments and failed to thrive in pure cultures at pH < 6 [45]. Studies confirmed that biochar elevated AOB *amoA* transcription levels [46], likely due to its ability to neutralize H^+^, raise soil pH, and create favorable conditions for AOB growth and activity, thereby enhancing nitrogen transformation efficiency [47]. Furthermore, biochar’s carbon-rich nature promoted carbon sequestration and transformation in agricultural soils, increasing CO_2_ flux and soil organic carbon (SOC) content. Elevated CO_2_ concentrations correlated with higher potential nitrification rates [48], while increased SOC enhanced both nitrification rates and microbial biomass carbon (MBC), positively influencing AOB *amoA* gene abundance. In contrast to AOB, biochar amendment did not significantly affect AOA *amoA* gene abundance in neutral and acidic soils. A study on paddy soils reported that long-term nitrogen fertilization markedly increased AOB *amoA* abundance and nitrification activity but had no significant impact on AOA *amoA* gene [49]. This phenomenon might arise from AOA’s higher tolerance to environmental fluctuations compared to AOB. These findings suggested that AOB *amoA* likely served as the primary driver of nitrification in biochar-amended acidic contaminated soils, a critical consideration for soil nitrogen cycle management.

These findings demonstrated that biochar could effectively mitigate Cd-induced inhibition of nitrification in acidic soils while having minimal impact on microbial communities in neutral soils. The study highlighted the importance of considering both soil type and environmental conditions when evaluating the efficacy of biochar as a soil amendment for improving soil health and productivity. Further research was needed to explore the long-term effects of biochar application on soil properties and microbial dynamics across diverse agro-ecological regions.

### 4.3. Response of Soil Bacterial Communities to Biochar in Varied pH Soils

The α-diversity of bacterial communities was higher in biochar-treated plots (SD-BC56d and YN-BC56d) compared to their respective untreated controls (SD-CK56d and YN-CK56d) (Figure 4), suggesting that biochar temporarily enhanced microbial diversity, consistent with previous studies [50]. In acidic soils, biochar addition significantly increased bacterial abundance compared to the untreated soils, which was consistent with Wang et al. [11] (pp. 627–638), who reported similar trends in Cd-contaminated acidic paddy soils. However, our study uniquely identified that this increase was driven by the enrichment of Nitrosomonadales (AOB) and *Gemmatimonadetes* (Figure 7). Conversely, in neutral soils, biochar application unexpectedly reduced bacterial abundance (Figure 5), a phenomenon diverging from most literature reporting neutral pH soils as favorable for microbial proliferation post-biochar [51]. We attribute this to fungal competitive dominance under biochar-induced pH shifts. Fungi exhibited a broader pH adaptability compared to bacteria, and they gained a competitive advantage under the altered pH conditions created by biochar, thereby suppressing bacterial proliferation [52].

Soil pH played a pivotal role in shaping microbial community structure following biochar amendment (Figure 6). In neutral soils, the disruption of original ecological equilibria likely relegated bacteria to subordinate niches, leading to reduced abundance and shifts in dominant taxa. The enrichment of Acidobacteria and suppression of Proteobacteria in neutral SD soils (Figure 5) contrasts with studies on neutral agricultural soils, where Proteobacteria typically dominate post-biochar [53]. Meanwhile, Zhu et al. [54] (p. 972300) demonstrated that biochar could modulate microbial community complexity in mining soils, reshape core microbial network structures, and increase rare taxa diversity. This suggested that Acidobacteria played a critical role in the ecological reconstruction of neutral soils treated with biochar. In contrast, in acidic soil, the microbial community exhibited greater compositional divergence under identical treatments, indicating unique responses to biochar in acidic conditions. Additionally, Methylomirabilota (NC10 phylum), exclusively detected in neutral SD soil, participates in denitrification by consuming H^+^ during nitrogen cycling. This process elevated soil negative charge, enhancing Cd^2^^+^ adsorption and indirectly reducing soluble Cd concentrations [55]. The synergistic action of Methylomirabilota and biochar facilitated Cd immobilization, improving soil Cd remediation stability. In neutral soils, the presence of specific microbial communities (such as Methylomirabilota) demonstrated the ability of biochar to recruit functional microorganisms that synergistically acted with its Cd adsorption mechanism. In field applications, biochar tailored to promote such taxa could reduce reliance on chemical amendments.

The functional traits of dominant bacterial taxa varied between the two soil types. In neutral soils, *Nitrospira* was significantly enriched following biochar amendment (Figure 7). Given *Nitrospira*’s nitrogen-transforming functionality in Cd-contaminated soils [56], biochar application likely stimulated nitrification in neutral SD soil. Conversely, in acidic red soil, Nitrosomonadales (AOB) and Gemmatimonadetes were enriched. Nitrosomonadales, as chemoautotrophs, drive ammonium-to-nitrite conversion [6], while Gemmatimonadetes decompose proteins to provide substrates for nitrification. Carbon source addition thus promoted organic decomposition and nitrification in acidic soils. Biochar-mediated environmental modifications enhanced bacterial metabolic functions, improving energy production, material cycling efficiency, and indirectly reinforcing Cd tolerance and self-remediation capacity [57]. This study observed biochar-induced enrichment in carbohydrate transport/metabolism, DNA replication/recombination/repair, and defense mechanisms. These pathways indicated strengthened microbial metabolic activity and stress resistance, synergistically enhancing heavy metal remediation alongside biochar’s Cd adsorption.

## 5. Conclusions

This study provided macro- and micro-scale insights into biochar application for Cd remediation and nitrification dynamics in soils with varied pH. Our analyses delineated fundamental dichotomies in remediation efficacy, with neutral matrices exhibiting sustainable Cd stabilization through ligand-assisted surface complexation mechanisms and selective enrichment of acidotolerant Acidobacteria populations. Conversely, acidic edaphic environments manifested progressive attenuation of passivation capacity, attributable to polyvalent metal ion dissolution dynamics, inherent proton abundance, and AOB-mediated nitrification-induced acidification cascades. Nitrification pathway specialization emerged as a critical pH-dependent divergence: neutral systems developed Nitrospiraceae-dominated nitrifying consortia (Nitrospirales ord. nov.), while acidic regimes selected for Nitrosomonadales-affiliated AOB assemblages. Reduced bacterial abundance but elevated Acidobacteria in neutral biochar-treated soil facilitated the assembly of a stress-resistant microbial community. Additionally, Methylomirabilota metabolism synergized with biochar to stabilize Cd remediation in neutral soils. These findings highlighted chemotactic selection of nitrifying communities by soil environments, with biochar mediating interactions between bacterial communities and Cd availability. These findings emphasized the necessity of pH-adaptive biochar dosing to reconcile long-term remediation stability with ecological functionality.

## Figures and Tables

**Table 1:** Basic physicochemical properties of the tested soil.

Soil Physicochemical Properties	Neutral Shandong Soil	Acidic Yunnan Soil
Clay content (%)	7.70	1.04
Sand content (%)	16.05	77.05
Silt content (%)	76.25	21.91
NO_3_^−^-N content (mg kg^−1^)	106.68	213.49
NH_4_^+^-N content (mg kg^−1^)	53.17	24.02
Available phosphorus (mg kg^−1^)	34.02	45.87
Organic matter (g kg^−1^)	28.15	109.01
pH	7.46	5.88
Moisture content (%)	2.66	13.11
Electrical conductivity (μS cm^−1^)	428	342
Total nitrogen (g kg^−1^)	1.00	4.07
Available Cd (mg kg^−1^)	0.37	1.57

**Table 2:** Basic properties of the tested BC.

Cottonseed hull biochar (BC)	Volatile matter (%)	Ash content (%)	pH	C (%)	H (%)	N (%)	S (%)	O (%)	Total Cd (mg kg^−1^)
	52.90	15.30	7.53	65.66	3.85	3.15	0.25	27.09	0.085

## Data Availability

The original contributions presented in this study are included in the article. Further inquiries can be directed to the corresponding authors.
